# Thermal Stability and Mechanical Behavior of Ultrafine-Grained Titanium with Different Impurity Content

**DOI:** 10.3390/ma16041339

**Published:** 2023-02-04

**Authors:** Kamil Majchrowicz, Agata Sotniczuk, Joanna Malicka, Emilia Choińska, Halina Garbacz

**Affiliations:** Faculty of Materials Science and Engineering, Warsaw University of Technology, Wołoska 141, 02-507 Warsaw, Poland

**Keywords:** Ti Grade 2, high-purity titanium, rolling, thermal stability, discontinuous yielding

## Abstract

Ultrafine-grained (UFG) commercially pure (Ti Grade 2) and high-purity (Ti 99.99%) titanium can be a good alternative to less biocompatible Ti alloys in many biomedical applications. Their severe plastic deformation may lead to a substantial increase of strength, but their highly refined microstructure show a lower thermal stability which may limit their range of applications. The purpose of this study was to investigate the effect of interstitial elements on the thermal stability of UFG Ti Grade 2 and high-purity Ti 99.99% processed by a multi-pass cold rolling to the total thickness reduction of 90%. The severely cold rolled Ti sheets were annealed at temperature in the range of 100–600 °C for 1 h and, subsequently, they were evaluated in terms of microstructure stability, mechanical performance as well as heat effects measured by differential scanning calorimetry (DSC). It was found that the microstructure and mechanical properties were relatively stable up to 200 and 400 °C in the case of UFG Ti 99.99% and Ti Grade 2, respectively. DSC measurements confirmed the aforementioned results about lower temperature of recovery and recrystallization processes in the high-purity titanium. Surprisingly, the discontinuous yielding phenomenon occurred in both investigated materials after annealing above their thermal stability range, which was further discussed based on their microstructural characteristics. Additionally, the so-called hardening by annealing effect was observed within their thermal stability range (i.e., at 100–400 °C for UFG Ti Grade 2 and 100 °C for UFG Ti 99.99%).

## 1. Introduction

A commercially pure (CP) titanium (Ti Grade 1–4) or a high purity (Ti 99.99%) titanium can be a good alternative to less biocompatible Ti alloys (e.g., the most commonly exploited Ti-6Al-4V) in many biomedical applications such as dental and maxillofacial implantology [1,2]. However, their mechanical properties may be insufficient to support oral loading in the recently developed miniaturized dental implants [3,4]. To increase the strength of CP Ti to a level of Ti alloys, its grain size refinement to an ultrafine-grained (UFG) regime (<1 µm) or a nanoscale (<100 nm) has been frequently applied in the last years [5]. This could be obtained by several plastic deformation techniques such as high-pressure torsion (HPT) [6,7,8], equal channel angular pressing (ECAP) [9,10,11], hydrostatic extrusion (HE) [12,13] or even a simple conventional cold rolling to large thickness reduction [14,15,16].

It is commonly known that the increasing amount of interstitial elements (such as oxygen or carbon) causes a significant strengthening of CP Ti and may enhance its thermal stability and recrystallization temperature [17,18]. Ouchi et al. [17] showed that even small additions of oxygen may affect its mechanical behavior, i.e., the increase of oxygen content from 36 to 830 ppm improved its strength by about 200 MPa (from around 200 to 400 MPa, respectively). The additional severe plastic deformation of CP Ti creates a chance to enhance its ultimate tensile strength close to the level of Ti-6Al-4V alloy (950 MPa [19]). Purcek et al. [10] were able to increase the strength of Ti Grade 2 and 4 by eight ECAP passes at 300 and 450 °C to 760 and 947 MPa, respectively. Moreover, the results obtained by Purcek et al. [10] and Greger et al. [11] showed that the ability of CP Ti for strain-hardening grows with lowering content of interstitial elements. The strength of Ti Grade 1, 2 and 4 after eight ECAP passes was enhanced by about 100, 43 and 20%, respectively, as compared to their as-received counterparts, indicating that the increasing impurities content hinders the grain refinement [10,11].

The severe plastic deformation of CP Ti may lead to a substantial increase of hardness and strength, but its highly refined microstructure shows a lower thermal stability which may limit their range of applications. The effect of applied strain on the thermal stability of UFG or nanograined CP Ti has been clearly presented by Huang et al. [8] who processed Ti Grade 2 discs by 10 turns of HPT. The short-time annealing for 10 min at 200 °C caused a drastic reduction of hardness by about 40% at the edge area (where the accumulated strain is the highest) while the hardness at the disc center (lower accumulated strain) was diminished by only 7%. Popov et al. [7] reported a slightly higher stability of mechanical properties for Ti Grade 2 processed by HPT at room temperature to the accumulated strain of ε = 7. They found a noticeable decrease of yield strength at 200–250 °C whereas the hardness was relatively stable up to 350 °C [7]. The thermal stability of Ti Grade 2 reported for other processing techniques enabling the accumulation of a lower total strain is usually higher. Li et al. [20] observed for Ti Grade 2 after a combined process of asymmetric rolling (to thickness reduction of 83%) and subsequent symmetric rolling (to 80%), that its hardness and tensile strength are stable up to 300 °C while, even after annealing at 400 °C for 30 min, the UFG structure with a grain size below 200 nm remained. Furthermore, Garbacz et al. [21] found that the hardness of Ti Grade 2 after hydrostatic extrusion to the accumulated strain of ε = 3.8 did not change significantly up to 400 °C. Finally, the thermal stability of CP Ti can be also affected by the increasing amount of interstitial elements. Dyakonov et al. [22] proved for Ti Grade 4 processed by ECAP at 200 °C that, despite the high accumulated strain of 8.4, its UFG structure and hardness were stable up to 400 °C.

Due to the high stored energy and high density of microstructural defects, the thermal stability of UFG Ti seems to be a critical aspect of their further usage in the biomedical field. This also results from the need of a sterilization process to which titanium implants are subjected before application, e.g., autoclave sterilization, ethanol immersion, gamma or ultraviolet irradiation [23,24]. The main method is the sterilization process in the autoclave which is performed at temperature ranging from 108 to 134 °C (usually 121 °C) and pressure above 1 bar [23,24]. Thus, the purpose of this study was to investigate the thermal stability and mechanical behavior of high-purity Ti 99.99% and Ti Grade 2 with UFG microstructure. A special emphasis was put on the UFG Ti 99.99%, for which microstructural and mechanical stability are scarcely reported in the literature. Due to the direct comparison of Ti 99.99% and Ti Grade 2 with the same processing history, it was possible to extract the influence of a different content of interstitial elements on the thermal stability and mechanical behavior of UFG Ti. To obtain the UFG structure, Ti 99.99% and Ti Grade 2 were subjected to the same conventional multi-pass cold rolling to the final thickness reduction of 90%. Then, they were annealed at temperature ranging from 100 to 600 °C and, subsequently, characterized in terms of microstructure stability by scanning and transmission electron microscopy and mechanical performance by microhardness and tensile tests. The described characterization was supplemented by calorimetric analysis that provided information if there are any noticeable heat effects which correspond to the microstructure evolution during annealing. Comparing annealing-induced changes for Ti with a different level of purity (Ti 99.99% and Ti Grade 2) after cold rolling to the same degree of deformation, allowed us to understand how interstitial elements affect thermal stability. Particular steps of designed procedure, precisely: (i) microstructure analysis, (ii) mechanical properties characterization and (iii) heat effects evaluation, have been described in detail in the Section 2.

## 2. Materials and Methods

### 2.1. Material Preparation

The exact chemical composition of the high-purity Ti 99.99% and technically pure Ti Grade 2 used in this study is listed in Table 1. Ti 99.99% was supplied in the form of ϕ14 mm as-drawn rod and, subsequently, annealed at 700 °C for 30 min to obtain an equiaxed microstructure having a grain size of about 45 ± 20 μm. Ti Grade 2 was also provided as ϕ14 mm extruded and annealed rod with a mean grain size of about 15 ± 6 µm. Then, they were machined into rectangular billets (10 mm × 10 mm × 50 mm) and subjected to multi-pass cold rolling to a final thickness reduction of 90% corresponding to total plastic strain of ε = 2.3. The as-rolled sheets were further annealed at temperature ranging from 100 to 600 °C for 1 h. All specimens were closed in quartz tubes filled with argon to avoid surface oxidation during heating.

### 2.2. Microstructure Characterization

The microstructure characterization of the as-rolled and annealed sheets was performed based on transmission electron microscope (TEM) and scanning electron microscope (SEM) observations using JEOL JEM-1200EX (JEOL Ltd., Tokyo, Japan) and Hitachi SU-8000 (Hitachi Ltd., Tokyo, Japan) microscopes, respectively. Thin foils for TEM investigation were prepared by cutting 3 mm diameter discs from a rolling direction–transverse direction (RD-TD) plane, which were then ground to a thickness of 0.1 mm and electropolished at a voltage of 35 V and temperature of 3 °C using a Struers TenuPol-5 system (Struers GmbH, Willich, Germany) and a Struers A3 electrolyte. These thin foils were also utilized for SEM observations which were conducted in the electron channeling contrast imaging (ECCI) mode using a backscattered electron (BSE) detector. SEM samples were additionally Ar^+^ ion polished using a Hitachi IM400 Ion Milling System (Hitachi Ltd., Tokyo, Japan) to obtain an adequate crystallographic contrast. Based on TEM and SEM images, the microstructure of the as-rolled and annealed sheets was quantified in terms of a mean grain size described by an equivalent diameter using MicroMeter software (v.086b) [26].

### 2.3. Mechanical Properties Characterization

The Vickers microhardness of the as-rolled and annealed sheets was firstly determined. It was measured on the RD-TD plane at a load of 200 g using an Innovatest Falcon 500 (Innovatest Europe BV, Maastricht, The Netherlands) hardness tester. In the next step, the selected samples were characterized in terms of yield strength (YS), ultimate tensile strength (UTS) and elongation to failure (A). The miniaturized tensile samples with a gauge length of 10 mm and a cross-section of 0.8 mm × 0.6 mm were machined by electrical discharge machining (EDM) along the RD. Tensile tests were conducted at an initial strain rate of 10^−3^ s^−1^ using a Zwick/Roell Z005 (Zwick GmbH & Co. KG, Ulm, Germany) testing machine equipped with a 1 kN load cell. Digital image correlation (DIC) system was used for strain measurements which has been described more in detail in [27,28]. Based on the obtained stress–strain curves, the strain-hardening rate Θ was calculated as Θ = dσ/dε where σ and ε are a true stress and true strain, respectively.

### 2.4. Heat Effects Measurements

Finally, the thermal stability of the severely deformed Ti 99.99% and Ti Grade 2 sheets was determined based on a differential scanning calorimetry (DSC) measurements using a TA Instruments Q2000 (TA Instruments, New Castle, DE, USA) calorimeter. Samples for DSC analysis were firstly isothermally heated at 40 °C for 3 min and then the temperature was increased up to 450 °C at a rate of 10 °C/min. As shown in the literature reports [7,22], the heating rate of 10–20 °C/min should allow for a precise differentiation of weak heat effects corresponding to microstructure evolution in the severely deformed titanium.

## 3. Results and Discussion

### 3.1. Microstructure of the As-Rolled Sheets

**Figure 1:**
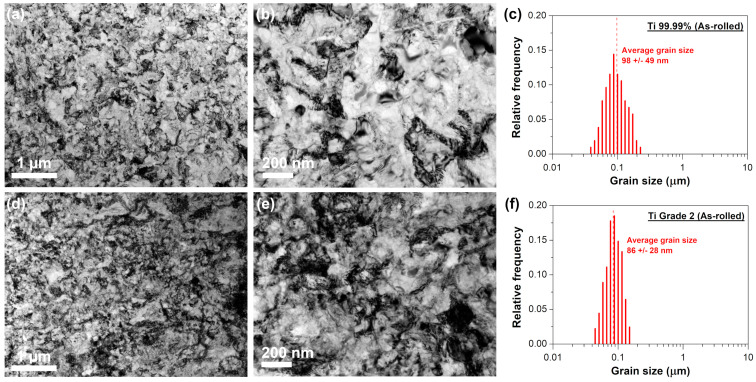
Microstructure of the as-rolled sheets: (**a**,**b**) Ti 99.99% and (**d**,**e**) Ti Grade 2 with the corresponding grain size distributions (**c**,**f**).

TEM observations revealed that multi-pass cold rolling to the thickness reduction of 90% resulted in a substantial refinement of both Ti 99.99% and Ti Grade 2 microstructure (Figure 1). It is claimed that the presence of refined grains in the heavily cold-rolled hexagonal titanium is associated to the mechanical twinning that allow to create lamellar structure during early stage of deformation [15,29]. Further deformation steps lead to lamellas breakdown by both formation of longitudinal dislocation walls and transverse dislocation boundaries. Described process gives a rise to formation of subgrains which, by the lattice rotations, can be subsequently transformed into the well-developed nanometric/ultrafine grains [14]. Based on the TEM analysis presented in our study, it was difficult to differentiate ultrafine grains in the as-rolled sheets (especially for Ti Grade 2) owing to the difficulties in the determination of the grain boundary position [30]. Similar findings were reported for Ti Grade 2, that was cold-rolled with the thickness reduction of 70% [31]. This problem is associated to the high dislocation density, typical for Ti subjected to the large plastic deformation [32], that blurred diffraction contrast related to the location of the grain boundary. Nevertheless, single well-defined ultrafine grains have been distinguished in the Ti Grade 2, having a mean size of about 86 ± 28 nm (Figure 1c). More well-developed ultrafine grains were visible for Ti 99.99% (Figure 1d,e). Their average size was estimated at 98 ± 49 nm (Figure 1f). This could be associated with the higher tendency of Ti 99.99% to undergo thermally-activated phenomena such as dynamic recovery. Performed calculations revealed that applying high rolling reductions could result in the significant deformation-induced heating that promote the recovery process. It is accepted that this phenomenon should be suppressed by the presence of impurities [10], which explain more developed ultrafine structure found for the Ti 99.99% as compared to Ti Grade 2.

### 3.2. Hardness Change during Annealing

**Figure 2:**
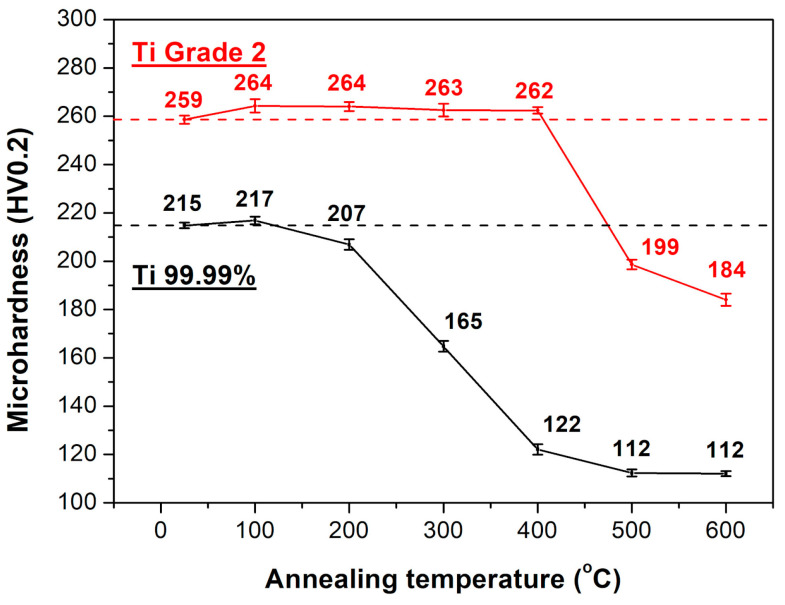
The variation of Vickers microhardness for Ti 99.99% and Ti Grade 2 after annealing at 100–600 °C.

The variation of Vickers microhardness for Ti 99.99% and Ti Grade 2 after annealing at temperature ranging from 100 to 600 °C for 1 h is presented in Figure 2. The hardness of the as-rolled sheets was 215 ± 1 and 259 ± 2 HV for Ti 99.99% and Ti Grade 2, respectively. The higher hardness of the Ti Grade 2 resulted from its more pronounced solid solution strengthening (due to the higher content of interstitial elements), grain boundary strengthening (lower grain size as shown in Figure 1c,f) as well as dislocation strengthening (higher dislocation density as presented in TEM images in Figure 1a,b,d,e). The annealing of Ti 99.99% at 100 °C caused a slight increase of hardness up to 217 ± 2 HV whereas at 200 °C it was changed down to 207 ± 2 HV. Further temperature increase resulted in a sharp decrease of hardness to a level of 112–122 HV after annealing at 400–600 °C. The microhardness of Ti Grade 2 was relatively stable up to 400 °C. In fact, it was slightly enhanced up to 262–264 HV. The drastic diminishment of hardness of Ti Grade 2 was observed after further annealing, i.e., it was reduced to 199 ± 2 and 184 ± 3 HV after heating at 500 and 600 °C, respectively. The Vickers microhardness measurements allowed us to define that mechanical properties of Ti 99.99% and Ti Grade 2 are relatively stable up to 200 and 400 °C, respectively. At higher annealing temperature, the hardness is drastically reduced and then stabilized at about 400 and 500 °C in the case of Ti 99.99% and Ti Grade 2, respectively. Thus, these experimental points have been selected for further microstructural analysis and tensile tests.

### 3.3. Microstructure of the Annealed Sheets

**Figure 3:**
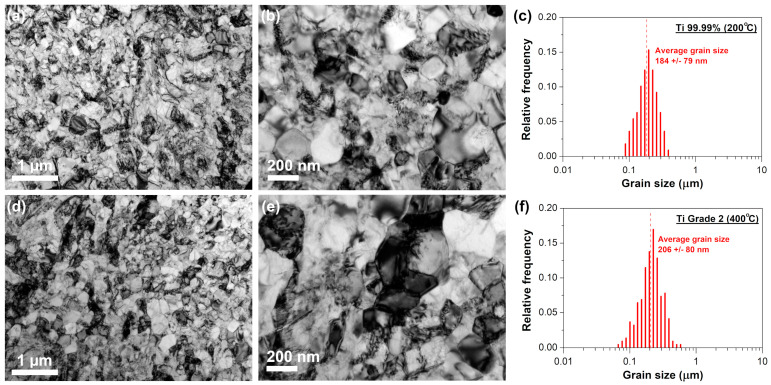
Microstructure of the annealed sheets: (**a**,**b**) Ti 99.99% at 200 °C and (**d**,**e**) Ti Grade 2 at 400 °C with the corresponding grain size distributions (**c**,**f**).

TEM analysis performed for the Ti 99.99% annealed at 200 °C and Ti Grade 2 annealed at 400 °C did not reveal noticeable thermally-induced grain growth (Figure 3). The lack of significant changes and stability of UFG structure in the case of selected annealed states, confirms the results of previously performed hardness tests (Figure 2). However, it can be noticed that, for both annealed materials, grain boundaries can be more easily distinguished compared to the as-rolled states. The existence of more developed UFG structure after annealing is related to the recovery process, which enables equilibration of grain boundaries by the annihilation of extrinsic grain boundary dislocations. Better-defined grain boundaries in the annealed samples enabled to precisely assess a mean size of ultrafine grains. Despite the significantly higher annealing temperature for Ti Grade 2, the average grain size was similar for both materials, i.e., 184 ± 79 and 206 ± 80 nm for Ti 99.99% and Ti Grade 2 (Figure 3c,f), respectively.

Recovery of the UFG structure is believed to be responsible for the unexpected slight hardening effect observed for the annealed Ti 99.99% and Ti Grade 2 (Figure 2), that is known in the literature as the “hardening by annealing” phenomenon [12,20,33,34]. The emission of new dislocations from the more equilibrium grain boundaries after annealing is more difficult and requires generation of higher stresses resulting in the strengthening of UFG materials [35,36,37]. It has also been shown that the occurrence of the hardening by annealing effect may be affected by the segregation of impurity atoms to grain boundaries [25,38,39,40]. According to the hardness results presented in Figure 2, the hardening effect seems to be more evident and stable for Ti with a higher content of interstitial elements. The hardness of UFG Ti 99.99% was increased by about 1% only after annealing at 100 °C, while for UFG Ti Grade 2, the increase of even 2.5% was evident up to 400 °C. It indicates that the hardening by annealing phenomenon can be obtained in the non-fully recovered Ti with a different content of interstitial elements but it is more effective for higher amounts of impurities. It seems to be consistent with findings of Renk et al. [38] who revealed by atom probe tomography of severely deformed 316L austenitic steel that the strengthening effect is not directly related to solute segregation. Instead, the solute atoms stabilize the ultrafine- or nanograined structure allowing for the occurrence of annihilation and relaxation processes necessary for hardening by annealing phenomenon.

**Figure 4:**
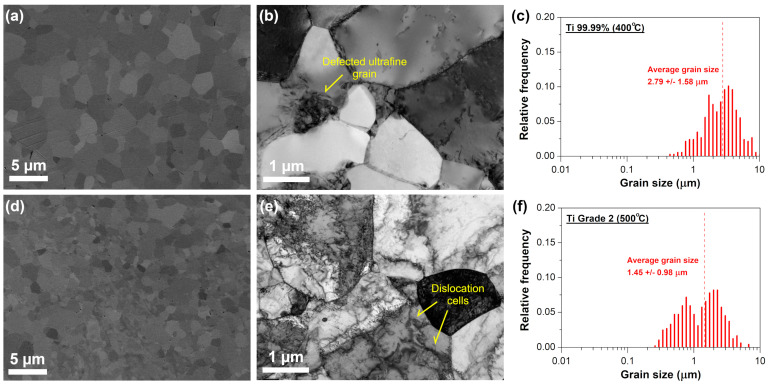
Microstructure of the annealed sheets: (**a**,**b**) Ti 99.99% at 400 °C and (**d**,**e**) Ti Grade 2 at 500 °C with the corresponding grain size distributions (**c**,**f**).

In order to gain the knowledge about the microstructure evolution during annealing at temperature above the microhardness stability, TEM analysis was performed for the Ti 99.99% and Ti Grade 2 annealed at 400 and 500 °C, respectively. Performed heat treatment resulted in the substantial rebuilt of microstructure and, for both annealed materials, single micrometric grains with well-defined grain boundaries were visible in TEM micrographs (Figure 4). In the case of Ti 99.99%, micrometric grains were virtually free of dislocations or contained rather single dislocations in their interiors with non-numerous dislocation tangles (Figure 4b). Apart from recrystallized micrometric grains, highly defected areas were still visible after annealing at 400 °C. Thereby, microstructure of heat-treated Ti 99.99% underwent partial recrystallization and contained a fraction of micrometric as well as ultrafine grains with high dislocation density which is clearly presented in the grain size distribution in Figure 4c. Similar, bimodal character of microstructure was observed for Ti Grade 2 after annealing at 500 °C (Figure 4d). Contrary to Ti 99.99%, a relatively high dislocation density was still present within the grain interiors, which indicates that its annihilation was suppressed by the presence of impurities. This finding correlates with the results of microhardness tests. For the cold-rolled Ti 99.99%, the heat treatment at 400 °C resulted in c.a. 43% decrease of hardness value while for Ti Grade 2, annealing at 500 °C brought almost two times smaller hardness reduction (c.a. 23%). Moreover, dislocation cellular substructures was observed in some micrometric grains of the annealed Ti Grade 2 (Figure 4e). This indicates that in the case of annealing of deformed Ti with a certain content of impurity atoms, formation of dislocation cells could be privileged compared to the dislocation migration to the well-defined high angle grain boundaries. Other differences between the microstructure of the annealed Ti 99.99% and Ti Grade 2 were related to the (i) fraction of ultrafine/micrometric grains, (ii) average diameter of micrometric grains and (iii) size distribution of micrometric grains which are clearly presented in the grain size distributions in Figure 4c and f obtained based on the SEM images. Despite the higher annealing temperature (500 °C), the average grain size for Ti Grade 2 was 1.45 ± 0.98 µm, whereas almost two times larger mean grain size was estimated for Ti 99.99% annealed at 400 °C, i.e., 2.79 ± 1.58 μm.

### 3.4. Tensile Properties of the Annealed Sheets

**Figure 5:**
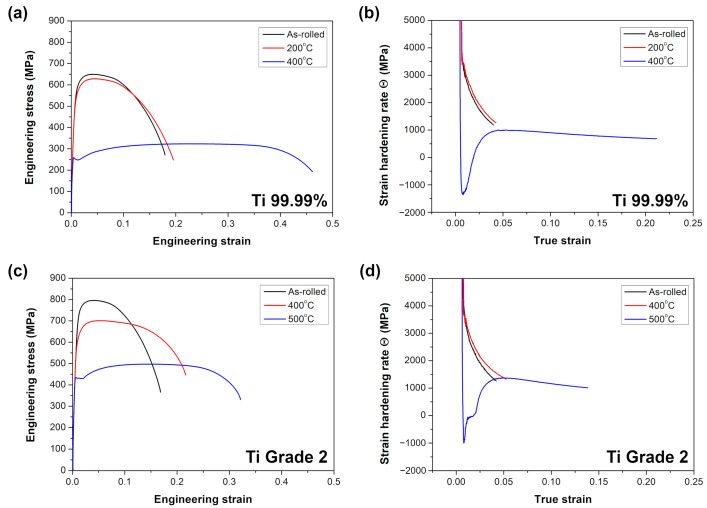
Stress–strain curves and strain-hardening rate Θ versus true strain for (**a**,**b**) Ti 99.99% and (**c**,**d**) Ti Grade 2.

The representative stress–strain curves for the as-rolled as well as annealed Ti 99.99% (at 200 and 400 °C) and Ti Grade 2 (400 and 500 °C) are shown in Figure 5 and the calculated mechanical parameters are summarized in Table 2. Similarly to the hardness measurements, the strength of the as-rolled Ti Grade 2 (YS = 600 ± 2 MPa, UTS = 798 ± 2 MPa) was significantly higher as compared to Ti 99.99% (YS = 481 ± 4 MPa, UTS = 644 ± 8 MPa). Further annealing within their microstructure stability range (i.e., at 200 and 400 °C for Ti 99.99% and Ti Grade 2, respectively) caused a slight decrease of YS and UTS down to: YS = 463 ± 6 MPa, UTS = 623 ± 6 MPa for Ti 99.99% and YS = 555 ± 2 MPa, UTS = 696 ± 5 MPa for Ti Grade 2. On the contrary to the hardness results, the YS of Ti Grade 2 was reduced by 7.5% after annealing at 400 °C, whereas the Vickers microhardness increased by 1.2%. This difference may be related to changes in the crystallographic texture. A typical texture of the heavily cold-rolled Ti sheets is a (0001)<10-10> texture with (0002) basal poles tilted of about 40° to TD [41,42]. As shown by Ghosh et al. [43], the annealing above 400 °C causes the strengthening of a basal texture intensity. The dominating deformation mechanism in Ti at room temperature is a dislocation <a> slip in the prismatic mode {10-10}<11-20> [44,45,46]. As shown by Chun et al. [47], the strengthening of basal texture intensity should decrease the Schmidt factor for prismatic <a> slip during compression along normal direction (the same direction as loading during hardness measurements) which hinders its activation and strengthens the material. At the same time, the prismatic <a> slip should be easier to activate during loading along RD (as in the tensile experiments) [48]. This explanation seems to be adequate since the difference between YS and hardness changes summarized in Table 2 were more evident for higher annealing temperature (at least 400 °C) for Ti Grade 2 as well as Ti 99.99%.

The annealing above the microstructure stability range (i.e., at 400 and 500 °C for Ti 99.99% and Ti Grade 2, respectively) caused a drastic reduction of YS and UTS while the elongation to failure was substantially improved (Figure 5 and Table 2). Surprisingly, a discontinuous yielding phenomenon has been observed for both materials although it is common that a coarse-grained Ti exhibits continuous yielding during tensile deformation [19]. The higher difference between upper and lower YS was noted for Ti 99.99% (UYS = 261 ± 3, LYS = 249 ± 3 MPa) as compared to Ti Grade 2 (UYS = 431 ± 5, LYS = 426 ± 3 MPa) while Ti Grade 2 exhibited a longer yield point elongation. The trend of the strain-hardening rate Θ for samples annealed above their microstructural stability range differed from that of the as-rolled ones as well as annealed within the thermal stability range (Figure 5b,d). The latter ones exhibited a typical monotonic slide of the strain-hardening rate; whereas, for Ti 99.99% annealed at 400 °C and Ti Grade 2 at 500 °C, the strain-hardening rate dropped at the beginning at a much faster rate and then it rapidly increased, indicating a typical discontinuous yielding. One of the first theories explaining the yield point phenomena was introduced by Cottrell and Bilby [49] who pointed out that it is attributed to the pinning of dislocation by solute atoms. This explanation was mostly utilized in the case of steels [50,51] but recently, it has been also used for a microcrystalline iron after different cooling processes [52]. Gao et al. [52] found the discontinuous yielding in the air-cooled iron while its water-cooled counterpart yielded continuously. The occurrence of discontinuous yielding has been explained by the locking of mobile dislocations by interstitial carbon and nitrogen atoms having enough time to form atmospheres around dislocations during slow air cooling while the rapid cooling in the water disabled the segregation of interstitials on mobile dislocations. Nevertheless, the aforementioned theory seems to be not adequate in our study since we observed the discontinuous yielding for both Ti Grade 2 as well as Ti 99.99% with a scarce solute atoms content. The yield point phenomenon has been recently found in some UFG or fine-grained pure materials even if their coarse-grained counterparts did not show such behavior [31,53,54,55,56]. The frequent explanation is based on Hahn’s theory [57] that the mobile dislocation density in the UFG materials is small after sufficient annealing treatment when it is possible to obtain fine grains almost free of dislocations. Moreover, it is also believed that the dislocation sources are inherently limited in the UFG materials [35]. Thus, a high stress is required to move those mobile dislocations or to form new dislocations to accommodate the plastic strain rate at the beginning of the tensile test. When the mobile dislocations are accumulated, the stress will decrease, leading to discontinuous yielding [31]. The accumulation of dislocations and their interactions after yielding results in a sudden increase of strain-hardening rate Θ [31,56] (as presented in the current study in Figure 5b,d). Another model was proposed by Sinclair et al. [58], who linked the yield point phenomena with the back-stress hardening coming from dislocation pile-ups and the dislocation forest hardening. The increasing and decreasing of the back stress at small strains (as a result of dislocation pile-ups at grain boundaries and then, their screening by the dislocations in the adjacent grain or on the other slip systems) is believed to be significantly large especially for a fine-grained materials [58]. Such explanation was covered by the results of Wu et al. [56] who observed the significant effect of the back stress on the mechanical behavior of CP Ti with a heterogenous lamella (bimodal) structure. Furthermore, Li et al. [55] and Xu et al. [31] showed that the yield point phenomenon in the CP Ti is much more evident for the fine-grained structure with the bimodal grain size distribution where the deformation of coarse grains occurs firstly, and then it is constrained by the smaller ultrafine grains declining to deform, resulting in the dislocation pile-ups at grain boundaries producing the back-stress. Nevertheless, the aforementioned different literature reports have a common factor which is the recovered fine-grained structure. It seems to be the key factor for obtaining the discontinuous yielding behavior since the mobile dislocations are mostly removed and the grain size is still sufficiently small which requires high stress to activate new dislocation sources. This explanation has been already covered by the results obtained by Kamikawa et al. [53] for high purity Al 99.99% with a grain size of about 3 µm and Tian et al. [59] for commercially pure Cu 99.9% with a grain size in the range from 0.5 to 3 μm. Therefore, it allows us to conclude that the discontinuous yielding observed for Ti 99.99% and Ti Grade 2 after annealing at 400 and 500 °C, respectively, results mostly from their fine-grained structure (Figure 4).

### 3.5. Heat Effects during Annealing

**Figure 6:**
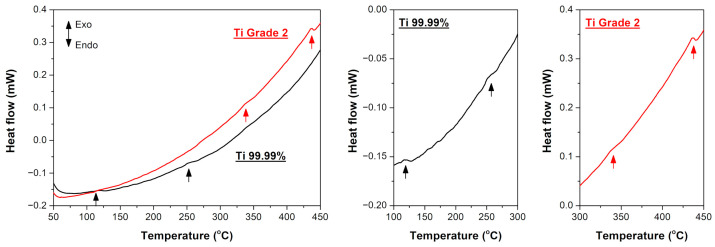
DSC curves representing heat flow as a function of annealing temperature for Ti 99.99% and Ti Grade 2.

DSC measurements have been performed in the final step. The obtained DSC curves representing heat flow change as a function of annealing temperature are presented in Figure 6. The as-rolled Ti 99.99% and Ti Grade 2 exhibited two peaks during heating, i.e., at about 120 and 250 °C for Ti 99.99% and at about 330 and 440 °C for Ti Grade 2. Similar behavior of UFG Ti Grade 2 was noted by Popov et al. [7] who found two peaks at 200 and 500 °C. The former one has been linked with the reduction of internal stresses and redistribution of dislocations while the latter one resulted from intensive grain growth. The microstructure evolution of both investigated materials presented in the current study (Figure 1, Figure 3 and Figure 4) also suggests that the first peaks (at 120 °C for Ti 99.99% and 330 °C for Ti Grade 2) are connected with the recovery process while the second peaks (at 250 °C for Ti 99.99% and 440 °C for Ti Grade 2) come from the substantial grain growth. It is consistent with other literature data which report that the significant exothermic processes at around 450 °C (or higher) are usually associated with the onset of recrystallization [22,60].

## 4. Conclusions

This work presented the effect of interstitial elements on the thermal stability and mechanical behavior of UFG Ti Grade 2 and high-purity Ti 99.99% processed by a multi-pass cold rolling to total thickness reduction of 90%. The main conclusions can be summarized as follows:

1. The microstructure and mechanical properties were relatively stable up to 200 and 400 °C in the case of UFG Ti 99.99% and Ti Grade 2, respectively. It was confirmed by DSC measurements that Ti 99.99% exhibited lower temperature of the onset of recovery process (at about 120 °C) and grain growth (250 °C) as compared to Ti Grade 2 (330 and 440 °C, respectively).
2. Surprisingly, the discontinuous yielding phenomenon occurred in both investigated materials after annealing above their thermal stability range, i.e., at 400 and 500 °C for Ti 99.99% and Ti Grade 2, respectively. It resulted mainly from their fine-grained microstructure with the average grain size of 2.79 and 1.45 μm for Ti 99.99% and Ti Grade 2, respectively.
3. The so-called hardening by annealing phenomenon was also observed for both investigated materials within their thermal stability range. The strengthening effect was more evident and stable for the higher content of interstitial elements. The hardness of UFG Ti 99.99% was increased by about 1% only after annealing at 100 °C, while for UFG Ti Grade 2 the increase of even 2.5% was evident up to 400 °C.

## Figures and Tables

**Table 1:** The content of impurities (including interstitial elements: C, H, N, O) in Ti 99.99% and Ti Grade 2 (in wt. %) [25].

Material	C	H	N	O	Fe
Ti 99.99%	0.0011	-	-	-	0.0011
Ti Grade 2	0.03	0.005	0.02	0.13	0.14

**Table 2:** Mechanical properties of the as-rolled and annealed Ti 99.99% and Ti Grade 2 (YS—0.2% offset yield strength, UTS—ultimate tensile strength, A—elongation to failure, HV—Vickers microhardness at a load of 200 g, ΔYS, ΔHV—changes in YS and HV after annealing).

Material	State	YS (MPa)	UTS (MPa)	A (%)	HV0.2	ΔYS (%)	ΔHV (%)
Ti 99.99%	As-rolled	481 ± 4	644 ± 8	17.9 ± 0.3	215 ± 1	-	-
	200 °C	463 ± 6	623 ± 6	18.4 ± 1.1	207 ± 2	−3.7	−3.7
	400 °C	261 ± 3/249 ± 3 *	326 ± 3	46.5 ± 0.7	122 ± 2	−45.7	−43.3
Ti Grade 2	As-rolled	600 ± 2	798 ± 2	16.4 ± 0.1	259 ± 2	-	-
	400 °C	555 ± 2	696 ± 5	21.3 ± 0.1	262 ± 1	−7.5	+1.2
	500 °C	431 ± 5/426 ± 3 *	495 ± 3	31.6 ± 2.9	199 ± 2	−28.2	−23.2

* UYS/LYS—upper/lower yield strength.

## Data Availability

The data presented in this study are available on request from the corresponding author.
